# Age prediction of hematoma using hyperspectral imaging (HSI)

**DOI:** 10.1007/s12024-025-01076-7

**Published:** 2025-09-11

**Authors:** S. Al-Arami, St. Lüdtke, J. Dreßler, C. Babian

**Affiliations:** 1 Institut für Rechtsmedizin Leipzig, Leipzig, Germany; 2 Institute for Visual & Analytic Computing, University of Rostock, Rostock, Germany

**Keywords:** Hyperspectral imaging, Hematoma, Age prediction, Machine learning, Forensic medicine

## Abstract

Hyperspectral imaging (HSI) analyzes the reflected light spectrum of an object, providing insights into its material composition. In this experimental, prospective study, standardized hematomas were created in subjects and observed over 21 days using a portable hyperspectral camera, aiming to correlate changes in the reflected light spectrum with hematoma age and enable an objective method for age determination of hematomas. In 25 young and healthy subjects, a hematoma was induced by injecting 3 ml of autologous blood on the volar side of the forearm. The hematomas were documented over a 7-day period in 24-hour intervals and then for an additional 14 days in 48-hour intervals using a portable HSI camera covering the wavelength range from 400 nm to 1000 nm. The datasets from the hematomas were normalized using the spectrum of unaffected skin. Lasso regression models were trained on the normalized data to identify age-dependent changes in the reflection spectra and to make age predictions for hematomas of unknown age. The different regression models were compared based on their predictive accuracy and then evaluated against the actual hematoma age. The best age prediction model based on HSI recordings achieved a mean prediction error of 3.35 days over a 21-day observation period.Expanding the data set and methodology, as well as combining them with other techniques and testing on naturally occurring hematomas of various sizes and locations, are necessary steps to transfer this method into practical application.

## Introduction

Blood extravasation into deeper layers of the skin (hematomas) is the most common consequence of blunt trauma. Determining the age of hematomas serves to narrow down the time of injury, reconstruct events, and verify the plausibility of statements made by victims, witnesses, or suspects. Previous methods for determining the age of hematomas primarily rely on visual inspection by more or less experienced examiners and their subjective evaluations of color and consistency. These conventional approaches are often inaccurate and can lead to false results. It has been shown that the subjective visual assessment of hematoma age based on photographs is unreliable, and the accuracy of the results does not increase with a higher degree of forensic expertise [3, 9, 10, 12, 15, 22]. Therefore, a legally sound, objective determination of hematoma age should be conducted in a standardized and examiner-independent manner.

There are approaches to estimate the age of hematomas using histology [1, 8], MRI examinations [4, 16], or with the aid of pulsed lasers and their photothermal absorption [13]; however, these methods often require invasive sampling or extensive technical and personnel resources, thus not allowing for a rapid, cost-effective, and flexible application in forensic practice.

Spectrophotometry represents another promising method, the potential of which has been demonstrated in various studies [2, 5–7, 9, 17, 18, 20, 25]. Therefore, the aim of this study was to establish a simple, mobile, objective, and reliable method for determining the age of hematomas using hyperspectral imaging (HSI). HSI analyzes the reflected light spectrum of an object, thereby allowing conclusions to be drawn about its material composition. While conventional digital cameras only capture color information in the visible range, the HSI camera enables the analysis of spectral data over a wide spectrum of light wavelengths, including the near-infrared range.

The optically perceptible color change during the healing process of a hematoma is a complex process and reflects the change in material composition within the hematoma. It depends on the initial pooling of extravasated blood [21], the diffusion of hemoglobin through upper skin layers [17], the overlay by melanin [23], as well as the recruitment of neutrophils and macrophages [8], their breakdown of extravasated hemoglobin [15], and the subsequent transport via lymphatic pathways [1]. During the breakdown of hemoglobin, oxyhemoglobin is converted to deoxyhemoglobin and then transformed by the enzyme heme oxygenase into biliverdin, which is subsequently converted to bilirubin by biliverdin reductase. The expression of these enzymes occurs in macrophages and neutrophils and follows a time-dependent course [11].

It has also been shown that the bilirubin concentration in the skin, measured with the help of a bilirubin meter, allows for inferences about the aging process [14]. This approach has not yet been developed into an age prediction model.

To determine the relationship between color changes during the healing process of a hematoma and its age, machine learning techniques (Lasso regression) were applied to make age predictions based on these models.

## Study design and methods

The study cohort comprised a total of 25 light-skinned subjects without known hematological disorders or prior trauma in the area of investigation, aged between 20 and 40 years, consisting of 15 male and 10 female participants. Each hematoma was created by subcutaneously injecting 3 ml of autologous blood into the volar side of the forearm.

HSI images were taken immediately before and after hematoma creation, as well as daily for the first week at 24-hour intervals. Additionally, images were taken every 48 h for the following two weeks to monitor the course of the hematomas over an extended period and document potential changes or developments. The HSI camera used was the Specim IQ^®^, manufactured by Specim, Spectral Imaging Ltd., based in Oulu, Finland. This system is based on a push-broom scanner that generates hyperspectral images in the range of 400 to 1000 nm with a spectral resolution of 7 nm (204 spectral bands). The effective pixel count is 512 × 512 pixels (x, y-axis), and the camera lens provides a field of view of 31 × 31 degrees.

**Fig. 1:**
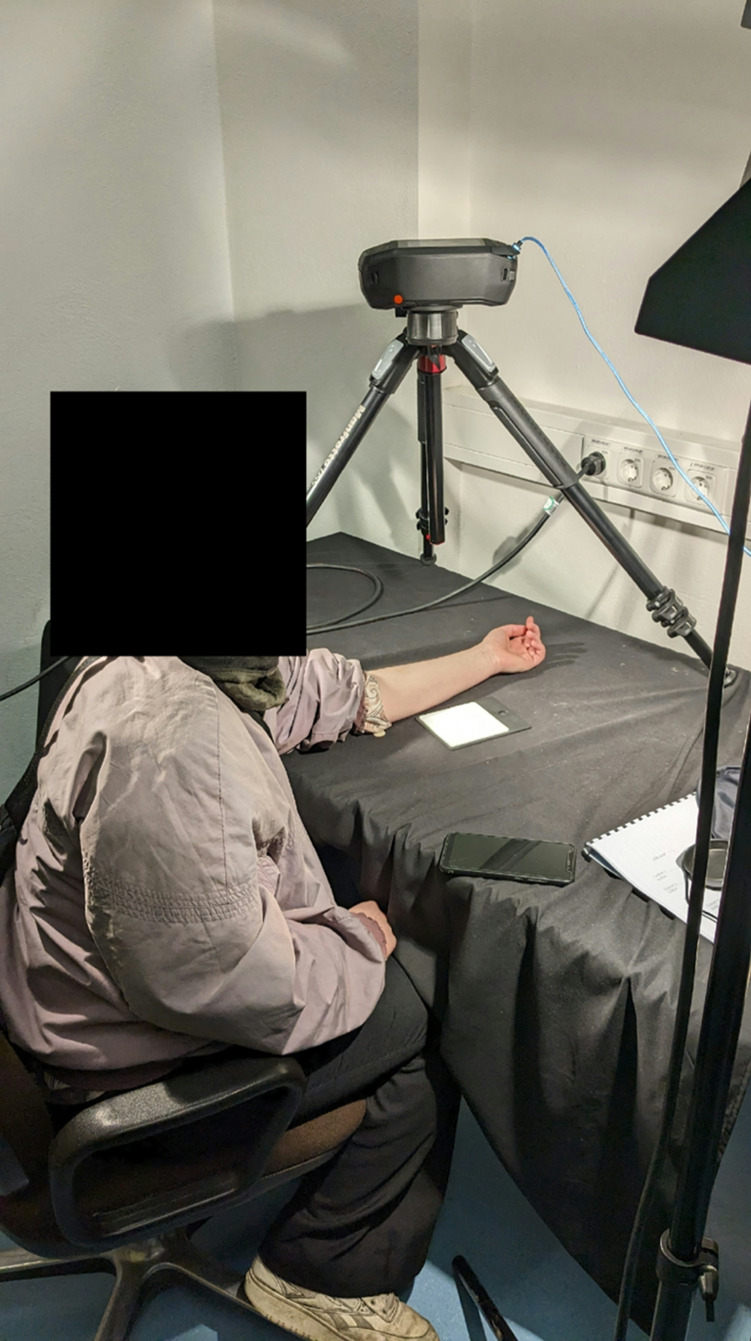
Camera setup

The camera was mounted on a tripod and set to a distance of 40 cm from the participant’s arm to ensure even illumination and focusing (see Fig. 1). Since the room could not be completely closed off from outside lighting, varying lighting conditions were present in the images. To address this, the hematoma area was slightly overexposed by two tungsten halogen broad-spectrum light sources (750 W each) and the integration time of the camera sensors was reduced (to 10 ms) to prevent oversaturation, resulting in more even lighting throughout the study, compensating for differences in outside light intensity due to recordings taken at various times of the year and day, thereby improving comparability.

The standard capture mode was used with a simultaneous white balance method at an integration time of 10 ms, in which the white reference tile, provided by Specim together with the Specim IQ^®^ camera system, is measured next to the target sample. This white reference tile is used to calibrate the Specim IQ^®^ camera by establishing a known reflectance standard, improving accuracy and consistency of the measurements across different lighting conditions and environments. The white balance is performed directly on the camera system after each recording using the software on the device.

**Fig. 2:**
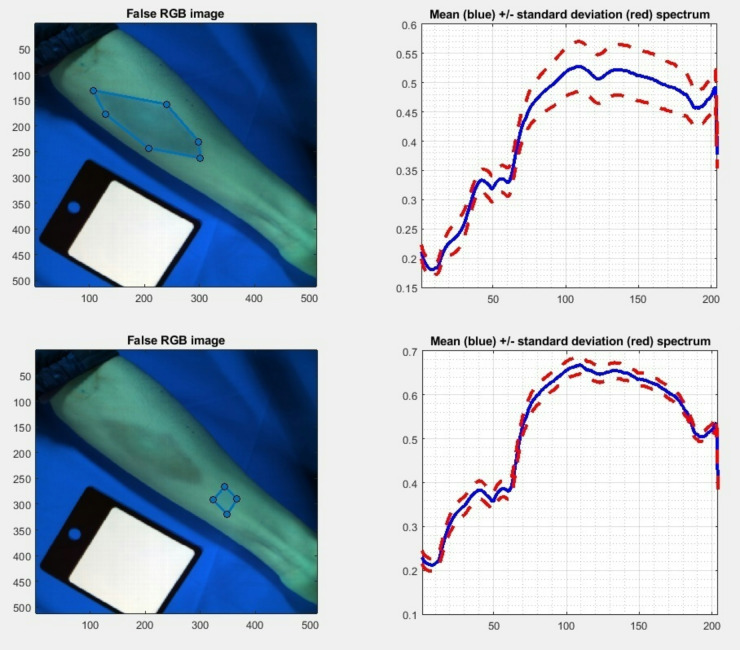
ROI selection using MatLab with hypertools3

The data were transferred to MATLAB to manually perform the ROI selection (Region of Interest) with the help of the MATLAB library Hypertools3 (see Fig. 2). The affected skin area, as well as an unaffected skin area in close proximity, were selected, and the average spectrum of the respective areas in the form of 204 spectral bands was extracted from the raw data.

Since the influence of spectral overlap due to the skin on the analysis is not known, we attempted to estimate and, if possible, minimize this effect by applying and comparing different normalization methods. As part of this approach, three different datasets were created, and all analyses were performed for each dataset: the first dataset included the raw data of the hematomas without normalization; in the second dataset, the spectrum of the unaffected skin was subtracted from the raw data; and in the third dataset, the spectrum of the unaffected skin was divided into the raw data.

A Lasso regression was conducted with each dataset to identify the spectral bands most relevant for age prediction. The programming language R was used for this, along with the glmnet and ggplot2 packages. Lasso regression is a form of linear regression that employs a regularization technique to reduce the number of features while preventing overfitting. Overfitting occurs when the model is too closely fitted to the training data and makes poor predictions on new data. This can lead to a model that becomes overly complex and learns the random variations in the training data instead of the actual patterns.

Lasso regression uses L1 regularization, which reduces the number of relevant features, resulting in a simpler and more robust model. Thus, irrelevant or redundant information is automatically eliminated. The outcome of the Lasso regression is a model in the form of a regression function, where a minimal amount of different spectral bands are used as features to achieve the highest possible accuracy in age predictions.

In addition to the three regression models, a more complex hierarchical model and a gender model were created. The hierarchical model is based on the observation of a biphasic course with an increase in hematoma intensity until approximately day 7, followed by a decrease in intensity thereafter. It consists of a sequence of several regression models: a first model trained with all data that assesses whether a hematoma is younger or older than 7 days. This is followed by the precise age estimation from a respective further model trained with the data up to day 7 and from day 7 onward.

The gender model consists of two regression models, each created with the datasets of all male and female participants, respectively. The models were tested using leave-one-person-out cross-validation. In this method, the respective model is trained with data from 24 of the 25 participants to predict the unknown age of the hematomas of the 25th participant. This process was repeated for each participant, and the predictions were compared with the actual age values to evaluate the performance of each model.

For each model, the total prediction error (root mean squared error, RMSE) was determined. This corresponds to the expected error of age estimation for the model with unknown hematomas.

## Results

The Lasso regression analysis identified the most relevant features as bands 23–26 and band 165, corresponding to wavelengths around 470 nm and 885 nm.

In the normalized data, the progression of intensities at these bands is more similar among the subjects, than in the raw data. The data normalized by division showed the most similar progression of intensity at these bands.

**Fig. 3:**
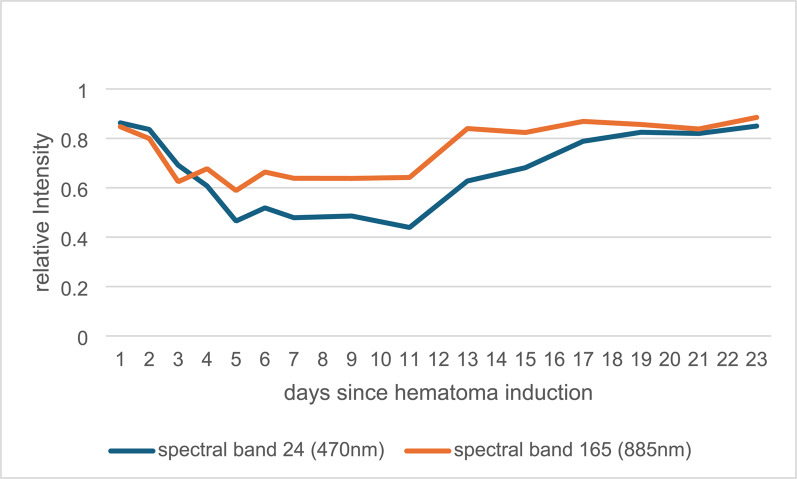
Intensity of relevant spectral bands of one subject during 23 days of investigation

Figure 3 shows the progression of the relevant spectral bands over the observation period for a male subject, based on data normalized by division. The intensity of band 24 (around 470 nm) exhibited a biphasic pattern, with a minimum between days 5 and 11, aligning with the observed decrease in intensity from day 7 onward. This change at 470 nm is most likely due to bilirubin absorption, as suggested by Mesli et al. [14], who demonstrated the relevance of bilirubin concentration in bruise aging using a bilirubinometer.

The intensity of band 165 at 885 nm shows a very early intensity minimum around days 3 to 5, so that the intensity begins to increase from this observation point and gradually approaches the intensity of normal skin over a period of about 21 days.

The model using the raw data achieved an RMSE value of 4.50 days through leave-one-subject-out cross-validation. In this case, young hematomas were assessed as too old, and old hematomas as too young. The differentiation of hematoma age appears to be more challenging for the model in hematomas overlaid by the spectrum of normal skin compared to increasing normalization.

The model using the data normalized by subtracting the skin spectrum improved the RMSE value to 4.24 days. This model can better distinguish young from older hematomas; however, hematomas are largely estimated to be too old.

The data normalized by dividing the skin spectrum achieved a further improvement of the RMSE value to 3.81 days. Thus, the dataset normalized by division yielded the best results among all three datasets.

Since the data normalized by division resulted in the most effective model, the hierarchical model and the gender model were trained based on this dataset. The hierarchical model achieved a further improvement of the RMSE value to 3.35 days, making it the best model. This model shows the best differentiation between old and young hematomas. For some subjects, deviations in predictions of less than 1 day were achieved. However, there were also subjects that could only be estimated with significant deviations by all tested models, especially in the earlier healing course.

The gender model showed a deterioration of the RMSE value to 3.91 days, although it is uncertain whether the approach itself or the significantly reduced data volume negatively affected the model’s quality.

Excluding images with visibly deviating lighting or angles from the training data of the best-performing model (Hierarchical Model) led to a slight deterioration in RMSE from 3.35 to 3.47. These deviations were identified post hoc based on clear visual differences, particularly on days with low ambient light despite our standardization approach. While overexposing the images with reduced sensor integration time generally achieved consistent lighting, certain recordings showed noticeable variation to the naked eye. These were selectively excluded to assess whether they impacted model performance; however, their removal did not improve results, suggesting the model’s relative robustness to minor lighting and angle inconsistencies.

**Fig. 4:**
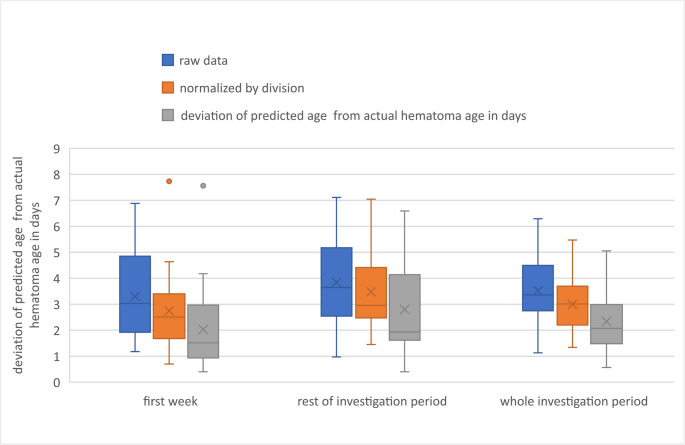
Deviations of the age predictions for the three models (raw data, normalized by division, and hierarchical model) from the actual age of the hematoma

Figure 4 shows the deviations of the predictions in days for the three models (raw data, normalized by division, and hierarchical model) from the actual age of the hematoma. This is represented for younger hematomas up to day 7, for older hematomas from day 7 onward, and for the entire observation period. The average absolute deviation of all predictions for each subject during the respective observation period was used for this purpose.

The predictions of all 25 subjects within the first week show deviations of (M = 3.30 SD = 1.66) for the raw data, (M = 2.74 SD = 1.48) for the data normalized by division, and (M = 2.03 SD = 1.57) for the hierarchical model. For the remaining observation period, during which recordings were made every 48 h, deviations of (M = 3.85 SD = 1.66) were achieved for the raw data, (M = 3.47 SD = 1.41) for the data normalized by division, and (M = 2.80 SD = 1.61) for the hierarchical model.

For the entire observation period, deviations of (M = 3.52 SD = 1.27) were found for the tested subject cohort for the raw data, (M = 3.00 SD = 1.07) for the data normalized by division, and (M = 2.33 SD = 1.1) for the hierarchical model. On average, across all subjects, the hierarchical model yields the best results for each hematoma age, with younger hematomas achieving the best age prediction.

## Discussion

For most subjects, normalizing the data based on the skin spectrum that overlays the hematoma spectrum improves prediction accuracy. Normalization through division yielded the best results among the normalization methods we tested. The best model was the hierarchically structured Lasso regression model based on the data normalized by division with the spectrum of unaffected skin, achieving a mean expected deviation (RMSE) of 3.35 days for previously unseen hematomas over an observation period of 21 days.

The accuracy of predictions in the first 7 days is higher than in the remaining observation period. It is unclear whether the reasons for this lie within the model itself or if they result from the fact that hematomas were documented in 48-hour intervals starting from day 7, thus providing fewer training data to the model. To improve results, a more frequent documentation of hematomas, especially after day 7, is advisable. Some recordings deviated from the 24-hour or 48-hour rhythm by 1–2 h, as it was not always possible for all subjects to adhere precisely to the scheduled times.

Due to the duration of this study over approximately three years, there were variances in the exposure of images due to different seasons, times of day, and weather conditions. Since it was impossible to assess the impact of these lighting conditions on the image captures, we overexposed all recordings with the help of external light sources and reduced the integration time of the camera sensors to ensure a more even lighting across the study. A standardized white balance was performed on each recording to ensure even better comparability and accuracy of the recorded spectral bands. After excluding images where the lighting conditions still deviated a little from the rest of the recordings, no improvement in results was observed.

Another limitation arises from the standardized creation of hematomas without real tissue trauma. This method, also used in the study by Neumayer B et al. [16], allows for high reproducibility but does not fully replicate the biological complexity of naturally occurring injuries. In real-world trauma, blood pooling in the extravascular space occurs gradually due to damaged blood vessels, as demonstrated in the histological studies by Barington K et al. [1] using a porcine model. This difference may influence the healing trajectory and, consequently, the spectral characteristics captured. Moreover, each hematoma in our study began with the same extravascular blood volume to ensure data comparability, as prior studies by Stam B et al. [21] and Scafide K et al. [19] have shown that initial volume significantly affects the healing process. Future studies should therefore include naturally occurring hematomas to better reflect the complexity of real trauma, and also examine a range of hematoma volumes to improve the generalizability and robustness of predictive models.

Due to easier analysis and reduced spectral overlap, all subjects in this study were selected to have light skin. As a result, the influence of increased melanin levels on recorded hematoma spectra, as examined in the study by Thavarajah D et al. [23], was not addressed. Additionally, the sample consisted exclusively of young, healthy individuals, which limits the generalizability of the findings. The spectral and healing characteristics of hematomas may differ significantly in older individuals or those with underlying health conditions. To improve the broader applicability of the model, future studies should include a more diverse sample in terms of age, skin tone, and health status.

The hematoma was induced at the same site (forearm) in all subjects, which presents another limitation of the study. While this approach improves standardization and reduces variability, it restricts the applicability of the findings to other anatomical locations. As the study by Randeberg L et al. [17] demonstrated, factors such as skin thickness and the diffusion of hemoglobin into different skin layers can significantly influence the recorded hyperspectral signal. Given that these physiological properties vary across body regions, the healing process and spectral characteristics of hematomas may differ accordingly. Therefore, future studies should include hematomas from multiple body sites to evaluate the robustness and generalizability of the method.

Since an average spectrum of the ROI was created for each hematoma and further analyses were conducted with these averaged values, the course of color heterogeneity within a hematoma, especially regarding the hematoma margin, was not included in the analyses, although macroscopic relationships can be inferred. In the first third of the healing process, sharper margins were observed, while more advanced healing showed softer transitions to the unaffected skin. Stam B et al. [20] showed that the analysis of these entities also allows for inferences about age.

Overall, it was observed that the intensity of the hematoma coloration recognizable to the naked eye varied more strongly than the intensity course of the relevant bands of reflected light. Thus, the color gradient was overall significantly darker in some subjects and very light in others, which showed no apparent influence on the model’s predictions.

Various studies have shown that the visual assessment of hematomas is unreliable and that the accuracy of these assessments does not increase with higher forensic expertise [3]. The accuracy of such predictions by forensic experts was under 40% in a meta-analysis of studies assessing hematomas in living children, showing poor inter-observer reliability [12].

So far, mainly basic research has been conducted on this topic, and only a small number of approaches and findings related to the age prediction of hematomas have been transformed into models that assign an age value to a hematoma, thus making an age prediction.

While the Lasso regression model used in this study was effective in managing high-dimensional spectral data, it inherently assumes a linear relationship between spectral features and hematoma age. However, the healing process of hematomas involves complex and non-linear biological mechanisms, which may not be fully captured by linear models. As a result, non-linear modeling approaches such as random forests, support vector machines, or neural networks could offer improved predictive performance, particularly in cases involving atypical healing patterns. A more detailed investigation into the sources of significant prediction errors is therefore necessary, especially for outliers and cases with irregular healing trajectories.

Notably, the best age prediction results based on spectral data were reported by Randeberg L et al. [17], who used reflectance spectroscopy in combination with a complex physical model rather than machine learning. Their method achieved age predictions with deviations of only 1 day over a 10-day observation period. However, the dataset consisted of just one spectral recording from each of 26 subjects, and the sample population included hospitalized patients undergoing medical procedures, most of whom were taking anticoagulants and had multiple pre-existing conditions. Despite these limitations, the high accuracy of the model highlights the potential of physically grounded approaches. A promising direction for future research could involve combining such physical models with advanced machine learning techniques to further enhance accuracy and generalizability across diverse patient groups and real-world scenarios.

Neumayer B et al. [16] estimated the age of hematomas using their MRI-based method, achieving an accuracy of 2.25 days deviation over a 14-day observation period. In this study, the hematomas were standardized by autologous blood injection in the thigh. Thus, this method achieves an average of about 1 day more accurate classification of hematomas within a 7-day shorter observation period, but requires significantly more technical and personnel resources.

In the context of these results, the prediction using the Lasso regression model with 3.35 days of deviation is less accurate but covers a larger observation period of 21 days within which the classification takes place. Furthermore, the method was tested using leave-one-subject-out cross-validation on over 300 spectral recordings of hematomas of different ages and from different subjects, thus relying on a larger database.

There is another approach by Tirado J and Mauricio D [24], which, similar to this study, assigns an age value to digital photographs of hematomas using machine learning techniques. For this purpose, a model was created using the training data that can classify the hematomas into one of seven classes. These classes are: a few hours to 2 days old, 3 days old, 4 to 6 days old, 7 to 12 days old, 13 to 17 days old, older than 17 days, and unaffected skin. The classification into these classes was achieved in the study with the best tested model with 97% precision, 99.5% specificity, and 97% sensitivity. These are very promising results, highlighting the potential of machine learning techniques in the age determination of hematomas. The classes cover a certain timeframe, increasing in size from class to class and covering more than 5 days in some instances. This approach is therefore only partially comparable in prediction quality with our method, which assigns a time value to each hematoma in hours. The deviations we achieved are sometimes smaller than the time periods covered by the assigned classes, which, however, does not necessarily indicate a better age prediction due to the different approaches. This approach, like all previous ones, also shares the commonality that accuracy decreases with increasing hematoma age.

So far, no method has been sufficiently tested and validated to establish it in practice in its current form. To translate the analysis of hematoma age based on specific light spectra into a practical method, expanding the underlying data is essential, as machine learning processes primarily yield better results with large datasets. Beyond a greater number of subjects, closer documentation of the healing process must also occur before applying it in practice, as the observation intervals limit accuracy. Various skin types and demographic groups must also be taken into account. Furthermore, combining the physical model used by Randeberg L et al. [17] with machine learning techniques could improve the methodology further, as machine learning primarily uncovers statistical relationships rather than necessarily real ones.

Since hyperspectral data also contains information about material composition, it offers a way to objectify hematoma findings and analyze images post-capture without loss of information, as with digital photography. The mobile HSI camera technology allows for the uncomplicated and mobile acquisition of such HSI data.

## Conclusion for practice

The hyperspectral analysis of reflected light from skin hematomas enables an investigator-independent and objective estimation of hematoma age, achieving an accuracy of 3.35 days over a 21-day monitoring period in this study. The use of Lasso regression provides a viable initial modeling approach; however, it assumes a linear relationship between spectral features and age. Given the biological complexity of hematoma healing, future work should explore non-linear models, which may improve predictive performance, particularly for atypical or older hematomas. Additionally, further analysis of prediction errors, especially in cases with delayed or abnormal healing, will be essential to refine model reliability.

Although the applied hyperspectral camera is portable and the biostatistical analysis is robust, the study was conducted under controlled conditions with relatively consistent lighting. In real-world forensic applications, however, lighting conditions can vary considerably and unpredictably, which may impact the quality and consistency of spectral data capture. While overexposure with reduced sensor integration time helped standardize lighting across the study, shielding ambient light more effectively in future studies may further improve data reliability. Additionally, the time required for processing and analyzing the spectral data remains a practical constraint; for this method to be viable in routine forensic use, the workflow must be streamlined to allow for faster and more efficient data processing in field or clinical settings.

Another limitation lies in the information loss due to averaging spectra across the region of interest (ROI), effectively treating the hyperspectral camera as a spectrometer rather than leveraging its spatial resolution capabilities. Integrating spatial-spectral analysis and incorporating domain-specific spectroscopic knowledge, such as known absorption behavior of tissue chromophores, may offer superior insights compared to machine learning alone and should be pursued in future research.

Furthermore, the study sample consisted exclusively of 25 young, healthy individuals with light skin, limiting the generalizability of the findings. Skin pigmentation, age, and underlying health conditions can significantly influence spectral characteristics and healing behavior. Expanding the participant pool to include individuals with diverse skin tones, ages, and medical backgrounds is critical to developing a broadly applicable model.

The artificial induction of hematomas through injection of autologous blood, while useful for standardization, does not fully replicate the complexities of real-world traumatic injuries. Naturally occurring hematomas may differ in terms of blood diffusion, tissue damage, and healing kinetics. Therefore, future studies should also include clinically or traumatically induced hematomas across various anatomical locations and volumes to better reflect forensic reality. As demonstrated by Randeberg L et al. [17], physical models may offer promising avenues for improved accuracy under more diverse and uncontrolled conditions and could be combined with machine learning techniques in the future.

In summary, while the current results demonstrate the potential of hyperspectral imaging as a forensic tool for dating hematomas, further methodological development and broader validation are necessary. By addressing the outlined limitations, expanding the dataset, improving modeling strategies, incorporating real-world cases, and accounting for technical variability, this approach could become an asset in both criminal and civil investigations.

## Data Availability

The datasets generated during and/or analyzed during the current study are available from the corresponding author on reasonable request.

## Appendix

See Figures 1, 2, 3 and 4. 

## Code availability

The code generated during the current study are available from the corresponding author on reasonable request.

## References

[1] Barington K, Jensen HE, et al. A novel, comprehensive, and reproducible Porcine model for determining the timing of bruises in forensic pathology. Forensic Sci Med Pathol. 2016;12(1):58–67. 26820283 10.1007/s12024-016-9744-6

[2] Bohnert M, Baumgartner R, et al. Spectrophotometric evaluation of the colour of intra- and subcutaneous bruises. Int J Legal Med. 2000;113(6):343–8. 11100429 10.1007/s004149900107

[3] Grossman S, Johnston A, et al. Can we assess the age of bruises? An attempt to develop an objective technique. Med Sci Law. 2011;51(3):170–6. 21905574 10.1258/msl.2011.010135

[4] Hassler E, Ogris K, et al. Contrast of artificial subcutaneous hematomas in MRI over time. Int J Legal Med. 2015;129(2):317–24. 25416961 10.1007/s00414-014-1124-8

[5] Hughes V, Ellis P, et al. The practical application of reflectance spectrophotometry for the demonstration of haemoglobin and its degradation in bruises. J Clin Pathol. 2004;57(4):355–9. 15047735 10.1136/jcp.2003.011445 PMC1770270

[6] Hughes V, Langlois N. Use of reflectance spectrophotometry and colorimetry in a general linear model for the determination of the age of bruises. For Sci Med Pathol. 2010;6(4):275–281. 10.1007/s12024-010-9171-z 20563889

[7] Hughes V, Langlois N. Visual and spectrophotometric observations related to histology in a small sample of bruises from cadavers. Forensic Sci Med Pathol. 2011;7(3):253–6. 21258881 10.1007/s12024-010-9221-6

[8] Kostadinova-Petrova I, Mitevska E, et al. Histological characteristics of bruises with different age. Open Access Macedonian J Med Sci. 2017;5(7):813–7. 10.3889/oamjms.2017.207 PMC5771278 29362602

[9] Langlois N. The science behind the quest to determine the age of bruises - a review of the english Language literature. Forensic Sci Med Pathol. 2007;3(4):241–51. 25869263 10.1007/s12024-007-9019-3

[10] Langlois N, Gresham G, et al. The ageing of bruises: A review and study of the colour changes with time. Forensic Sci Int. 1991;50(2):227–38. 1748358 10.1016/0379-0738(91)90154-b

[11] Langlois N, Olds K, et al. Heme oxygenase-1 and Heme oxygenase-2 expression in bruises. Forensic Sci Med Pathol. 2015;11(4):482–7. 25772118 10.1007/s12024-015-9660-1

[12] Maguire S, Mann MK, et al. Can you age bruises accurately in children? A systematic review. Arch Dis Child. 2005;90(2):187–9. 15665179 10.1136/adc.2003.044073 PMC1720275

[13] Marin A, Hren R, et al. Pulsed photothermal radiometric depth profiling of bruises by 532 Nm and 1064 Nm lasers. Sensors. 2023;23(4):2196. 36850795 10.3390/s23042196 PMC9965129

[14] Mesli V, Le Garff E, et al. Determination of the age of bruises using a bilirubinometer. Forensic Sci Int. 2019;302:109831. 31255841 10.1016/j.forsciint.2019.05.047

[15] Mosqueda L, Burnight K, et al. The life cycle of bruises in older adults. J Am Geriatr Soc. 2005;53(8):1339–43. 16078959 10.1111/j.1532-5415.2005.53406.x

[16] Neumayer B, Hassler E, et al. Age determination of soft tissue hematomas. NMR Biomed. 2014;27(11):1397–402. 25208978 10.1002/nbm.3202

[17] Randeberg L, Haugen O, et al. A novel approach to age determination of traumatic injuries by reflectance spectroscopy. Lasers Surg Med. 2006;38(4):277–89. 16538661 10.1002/lsm.20301

[18] Randeberg L, Larsen E, et al. Characterization of vascular structures and skin bruises using hyperspectral imaging, image analysis and diffusion theory. J Biophotonics. 2010;3(1–2):53–65. 19739145 10.1002/jbio.200910059

[19] Scafide K, Sheridan D, et al. Evaluating change in bruise colorimetry and the effect of subject characteristics over time. Forensic Sci Med Pathol. 2013;9(3):367–76. 23839662 10.1007/s12024-013-9452-4

[20] Stam B, van Gemert M, et al. Can color inhomogeneity of bruises be used to Establish their age? J Biophotonics. 2011;4(10):759–67. 21595043 10.1002/jbio.201100021

[21] Stam B, van Gemert M, et al. How the blood pool properties at onset affect the Temporal behavior of simulated bruises. Med Biol Eng Comput. 2012;50(2):165–71. 22261914 10.1007/s11517-012-0860-5 PMC3272227

[22] Stephenson T, Bialas Y. Estimation of the age of bruising. Arch Dis Child. 1996;74:53–5. 8660049 10.1136/adc.74.1.53 PMC1511603

[23] Thavarajah D, Vanezis P, et al. Assessment of bruise age on dark-skinned individuals using tristimulus colorimetry. Med Sci Law. 2012;52(1):6–11. 22041123 10.1258/msl.2011.011038

[24] Tirado J, Mauricio D. Bruise dating using deep learning. J Forensic Sci. 2020;66:336–46. 32991003 10.1111/1556-4029.14578 PMC7821214

[25] Yajima Y, Funayama M, et al. Spectrophotometric and tristimulus analysis of the colors of subcutaneous bleeding in living persons. Forensic Sci Int. 2006;156(2–3):131–7. 16410163 10.1016/j.forsciint.2003.09.022

